# Intervalence charge transfer of Cr^3+^-Cr^3+^ aggregation for NIR-II luminescence

**DOI:** 10.1038/s41377-023-01219-x

**Published:** 2023-07-25

**Authors:** Shengqiang Liu, Jingxuan Du, Zhen Song, Chonggeng Ma, Quanlin Liu

**Affiliations:** 1 Beijing Municipal Key Laboratory of New Energy Materials and Technologies, School of Materials Sciences and Engineering, University of Science and Technology Beijing, Beijing, 100083 China; 2 School of Optoelectronic Engineering & CQUPT-BUL Innovation Institute, Chongqing University of Posts and Telecommunications, Chongqing, 400065 China

**Keywords:** Inorganic LEDs, Optical materials and structures

## Abstract

The increasing demand for high-contrast biological imaging, non-destructive testing, and infrared night vision can be addressed by the development of high-performance NIR light-emitting materials. Unlike lanthanide (Ln^3+^) with sharp-line multiplets and isolated Cr^3+^ with NIR-I emission, this study reports the first-ever NIR-II broadband luminescence based on the intervalence charge transfer (IVCT) of Cr^3+^-Cr^3+^ aggregation in gallate magentoplumbite. In particular, LaMgGa_11_O_19_:0.7Cr^3+^ exhibits dual-emission (NIR-I, 890 nm and NIR-II, 1200 nm) with a full width at half maximum (FWHM) of 626 nm under 450 nm blue LED excitation. Moreover, this dual-emission exhibits anti-thermal quenching behavior (432% @ 290 K), attributed to the energy transfer among multiple Cr^3+^ centers. Cryogen absorption spectra, lifetimes decay (2.3 ms), and electron paramagnetic experiments reveal the NIR-II luminescence of the Cr^3+^-Cr^3+^ → Cr^2+^-Cr^4+^ IVCT transition. The application of LaMgGa_11_O_19_:0.7Cr^3+^ in NIR-II biological imaging as an optical contrast agent, non-destructive testing, and night vision is demonstrated. This work provides new insights into broadband NIR-II luminescence under UV-NIR excitation based on the IVCT of Cr^3+^-Cr^3+^ aggregation.

## Abstract (web summary)

Intervalence charge transfer (IVCT) for NIR-II luminescence was first reported in heavy Cr^3+^ doped LaMgGa_11_O_19_:Cr^3+^. The luminescence exhibits anti-thermal quenching behavior (432% @ 290 K), capable of NIR-II spectroscopy applications.

## Graphical abstract

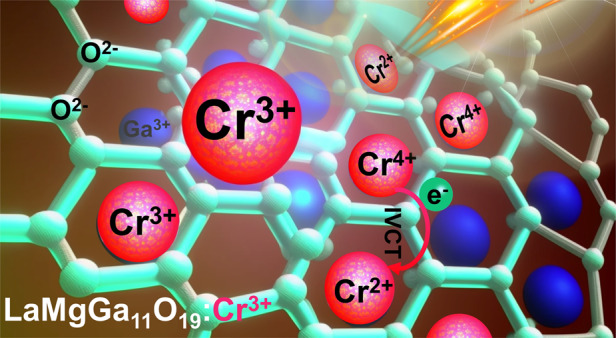

## Introduction

Near-infrared (NIR) luminescence imaging technology, particularly when working in tandem with other modalities to achieve real-time signal acquisition, is a practical tool for in vivo diagnostics and drug delivery^1–3^. Light-mediated photodynamic (PDT) and photothermal therapies (PTT) are both facilitated through the utilization of the NIR biological imaging window^4^. The research pertaining to luminescence imaging predominantly concentrates on high-performance NIR light-emitting contrast agents that demonstrate excitation and luminescence within the biological imaging window^5,6^. When contrasted with the conventional first imaging window (NIR-I, 750–950 nm), luminescent contrast agents that operate within the second imaging window (NIR-II, 1000-1800 nm) exhibit lower tissue absorption and scattering coefficient. This, in turn, enables a larger probing depth, lower autofluorescence, and a higher imaging signal-to-noise ratio. Besides, NIR phosphor-converted light-emitting diodes (pc-LEDs) are receiving increasing interest in fields such as non-destructive testing, plant cultivation, and night vision^7,8^. In 2016, Osram reported the first NIR pc-LED, SFH4735, covering 650–1050 nm^9^. However, the output power in the NIR region is typically low in empirical terms (16 mW @ 350 mA), and the luminescence spectrum generally encompasses the NIR-I region with a narrow full width at half maximum (FWHM). Consequently, NIR light-emitting phosphor is the key enabler in integrating this compact NIR device for spectroscopy analysis, particularly within the NIR-II region.

Inorganic NIR light-emitting converters, which consist of lanthanide Ln^3+^ elements, have been extensively studied and reported to be exceptional contrast agents and modulators that utilize linear emission, upconversion, and downconversion^10–12^. The distinctive energy-level configuration of Ln^3+^ allows for effortless spectral and temporal discrimination, making it ideal for analyzing subcellular processes. However, due to the shielding of *f*-*f* transition by outer-shell electrons, Ln^3+^ typically exhibits sharp multiplets emissions ranging from ultraviolet to NIR. Consequently, optical contrast agents that relay on Ln^3+^ are inevitably limited by the tunability of the spectrum and may introduce visible background signals. Besides, due to the broadband vibration absorption of organic functional groups^8,13^, it is unsuitable for nondestructive testing of molecular structures and chemical components. Cr^3+^ ([Ar]3d^3^) as an ideal NIR light-emitting activator, has been extensively explored in garnet^14,15^, borophosphate^16,17^, spinel^18^, pyroxene^19–21^, and double-perovskite^22–24^. However, the luminescence of Cr^3+^ in octahedral site is typically located in the NIR-I region, as illustrated by the Tanabe-Sugano diagram^25^. The presence of Cr^4+^ ([Ar]3d^2^) is capable of extending the emission to the NIR-II region^26,27^, but the efficiency of Cr^4+^ is typically low due to poor luminescence thermal quenching at room temperature.

Luminescence-molecule aggregation inducing light-emitting was first reported in 1-methyl-1,2,3,4,5-pentaphenylsilole by Tang et al., which provides valuable insight into the anti-quenching behavior of aggregation-caused luminescence^28,29^. In the case of transition-metal ions (e.g. Mn^2+^, Cr^3+^) doped phosphors, the radiative transition typically originates from an isolated luminescence activator, and luminescence concentration quenching may occur with dopant aggregation^30^. Our group previously reported the suppressed concentration quenching in Cr^3+^/Mn^2+^ doped β-Ca_3_(PO_4_)_2_-type compound due to the structural confinement effect^31,32^. However, due to the significant structural rigidity of β-Ca_3_(PO_4_)_2_, regulating luminescence properties through crystal-field engineering is challenging. In fact, as the concentration of dopants such as Mn^2+^/Cr^3+^ increases, neighboring ions may form dimers or clusters, resulting in strong interactions between the unshielded 3d electrons, such as magnetic interactions and intervalence charge transfer (IVCT)^33–36^. For NIR spectroscopy applications, we were the first to report the broadband NIR-II emission based on IVCT of Cr^3+^-Cr^3+^ aggregation in gallate magentoplumbite.

The magentoplumbite-type structure with AB_12_O_19_ formula is commonly found in chondritic meteorites and provides five independent B crystallographic sites (e.g. Mg, Ni, Al, Ga, In) for Cr^3+^ incorporation, which is favorable for achieving tunable NIR-I emission^37–39^. Due to its long decay lifetime (1.1-2.4 ms) in the Sr(Al,Ga)_12_O_19_:Cr^3+^ host, Rajendran et al., attributed the broadband NIR-I emission (740-820 nm) to Cr^3+^-Cr^3+^ magnetic interaction^40^. In this study, we explore the NIR-II IVCT luminescence (1200 nm) of Cr^3+^-Cr^3+^ → Cr^2+^-Cr^4+^ in a heavy Cr^3+^ doped LaMgGa_11_O_19_ magentoplumbite host. We conducted detailed investigations of the crystal structure, cryogen absorption spectra, lifetime decay, X-ray absorption near-edge structure (XANES), and electron paramagnetic resonance (EPR) experiments to identify the NIR-II luminescence of the IVCT. Notably, LaMgGa_11_O_19_:0.7Cr^3+^ exhibits a super-broad dual-emission (890 and 1200 nm) with a FWHM of 626 nm. Besides, this emission presents anti-thermal quenching behavior (432% @ 290 K) due to efficient energy transfer (ET) among multiple luminescence centers. This work offers valuable insights into NIR-II emission based on IVCT of Cr^3+^-Cr^3+^ aggregation, which is capable of high-contrast in vivo imaging and broadband pc-LEDs applications.

## Results

### Crystal structure and phase identifications

**Fig. 1:**
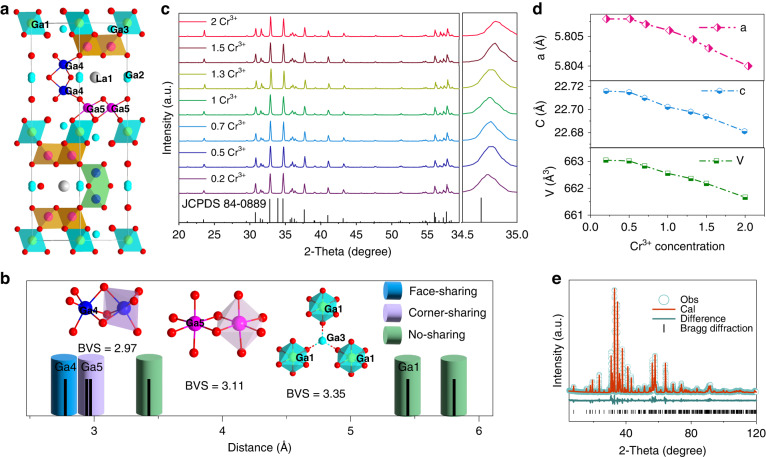
Crystal structure and XRD patterns **a** Crystal structure and Ga-O octahedra of LaMgGa_11_O_19_. **b** Cation distance and sharing method of Ga-Ga polyhedron. **c** XRD patterns of LaMgGa_11-*x*_O_19_:*x*Cr^3+^ (*x* = 0–2). **d** Lattice parameters and volume versus Cr^3+^ concentration. **e** Rietveld refinement of LaMgGa_11_O_19_:0.7Cr^3+^ sample

LaMgGa_11_O_19_ is crystalized to a hexagonal magentoplumbite-type structure (*P 6*_*3*_/*mmc*, 194) with five independent Ga crystallographic sites, as illustrated in Fig. 1a. The Ga1 2*a*, Ga4 4 *f*, and Ga5 12*k* are six-oxygen coordinated and capable of facilitating the NIR emission of octahedral Cr^3+^ ion^37^. In particular, Ga4-O octahedra are connected to each other via face-sharing with the shortest Ga-Ga distance of 2.80 Å, while the Ga5-O octahedra share edges with a Ga-Ga distance of 2.94 Å. The Ga1-O octahedra are separated by the Ga3 site, resulting in the longest Ga-Ga distance of 5.81 Å, as depicted in Fig. 1b. Consequently, strong interactions are expected to occur with increasing Cr^3+^ concentration in the Ga4 site, and the Cr^3+^-Cr^3+^ aggregation may result in pair luminescence due to the short cation distance. Ga3 4 *f* site is coordinated with four oxygen, while the Ga2 4*e* deviates from trigonal bipyramidal symmetry and splits into two mirror-symmetric pseudo-tetrahedra. According to our previous work^37^, Mg^2+^ selectively occupies the Ga1 and Ga3 sites along the (001) plane. Figure 1c illustrates the XRD patterns of LaMgGa_11-*x*_O_19_:*x*Cr^3+^, and all the peaks are indexed to a standard card #JCPDS84-0889, indicating successful incorporation of Cr^3+^ into the hexagonal matrix. As the Cr^3+^ concentration increases, the diffraction peaks slightly shift to higher angels with lattice shrinkage (Fig. 1d), which is attributed to the substitution of smaller Cr^3+^ (0.615 Å, CN = 6) for Ga^3+^ (0.62 Å, CN = 6). The structural refinement pattern (Fig. 1e) indicates that Cr^3+^ selectively substitutes the Ga4 and Ga5 crystallographic sites with 20% and 11% occupations, respectively, while only 5% occupies the Ga1 site (Table S1). Furthermore, the bond valence sum (BVS) of the Ga4 (+2.97) and Ga5 (+3.11) sites are both within +3, whereas the Ga1 site is under-bonded with a BVS value of +3.35, which is significantly larger than +3. This suggests that Cr^3+^ is incorporated into the hexagonal lattice by first occupying the Ga4 and Ga5 sites, which brings the BVS value closer to +3. These findings further indicate that cation aggregation may occur in the Ga4 site due to the selective occupation of Cr^3+^. Figure S1 displays the microscopic morphology images with an average size of 3 μm, and the EDS elemental mappings reveal a uniform component distribution.

### NIR-II luminescence properties

**Fig. 2:**
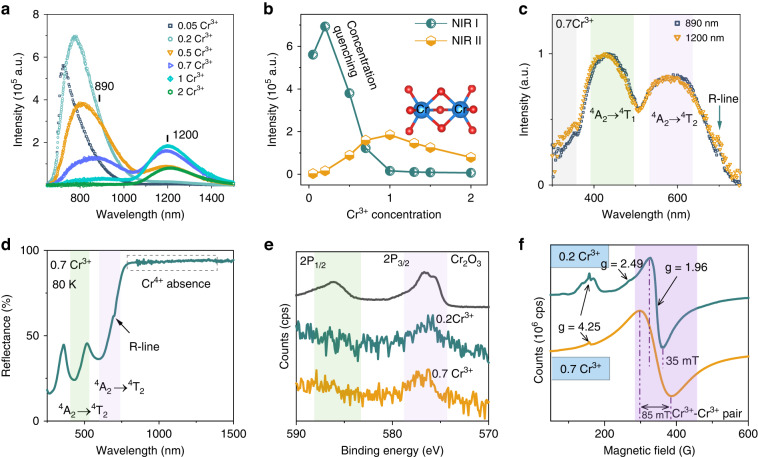
NIR-II photoluminescence properties and valence characterization of Cr. **a** PL spectra of LaMgGa_11-*x*_O_19_:*x*Cr^3+^ (*x* = 0–2) under 440 nm excitation. **b** Luminescence intensities of NIR-I and NIR-II versus Cr^3+^ concentration. **c** PLE spectra of LaMgGa_11_O_19_:0.7Cr^3+^ monitoring at 890 and 1200 nm. **d** Cryogenic (80 K) UV-Vis-NIR diffuse reflectance curve. **e** XPS curves of Cr_2_O_3_, LaMgGa_11_O_19_:0.2Cr^3+^, and LaMgGa_11_O_19_:0.7Cr^3+^ samples. **f** EPR curves of LaMgGa_11_O_19_:0.2Cr^3+^ and LaMgGa_11_O_19_:0.7Cr^3+^ samples

Figure 2a demonstrates the PL spectra of LaMgGa_11-*x*_O_19_:*x*Cr^3+^ at room temperature. Under excitation at 440 nm, the 0.2Cr^3+^ sample exhibits a proficient broadband NIR-I emission spanning from 650 to 1000 nm, which is attributed to the ^4^A_2_ → ^4^T_2_ spin-allowed transition of isolated Cr^3+^^37^. With an increase in Cr^3+^ concentration, the intensity of NIR-I luminescence continuously decreases due to concentration quenching (Fig. 2b). Furthermore, these emission peaks exhibit continuous red-shift from 720 to 890 nm due to the efficient ET among multiple isolated Cr^3+^ centers. The detailed discussion of this phenomenon can be found in the supplementary information and Figs. S2–S4^37^. In addition to the NIR-I emission, a broadband NIR-II emission (1200 nm) emerges with heavy Cr^3+^ incorporation (>0.5). The Cr^3+^ concentration shows little effect on NIR-II emission peaks, but the emission intensity first increases and then quenches. Notably, at 0.7 Cr^3+^, LaMgGa_11_O_19_ displays a dual-emission (890 and 1200 nm) with a FWHM of 626 nm. Furthermore, a broadband excitation ranging from 300 to 750 nm is presented by monitoring these two emissions (Fig. 2c), which are attributed to the ^4^A_2_ → ^4^T_1_ and ^4^A_2_ → ^4^T_2_ spin-allowed transitions of the isolated Cr^3+^. Compared with the NIR-I, the NIR-II emission features a much larger Stokes shift (622 nm), indicating a lower secondary inner filter effect, as shown in Fig. S5. Additionally, there is no overlap between excitation and emission spectra of NIR-II luminescence, indicating high imaging contrast and detection sensitivity, which can be utilized as optical contrast agents. Although the ^3^A_2_ → ^3^T_2_ inter-configurational transition of Cr^4+^ also presents tunable broadband NIR-II emission, the excitation signal of Cr^4+^ cannot be observed monitoring at 1200 nm, validating that the NIR-II emission is unrelated to Cr^4+^ impurity. The normalized Cr-K edge XANES of 0.7 Cr^3+^ sample was presented in Fig. S6. Both LaMgGa_11_O_19_:0.7Cr^3+^ and Cr_2_O_3_ exhibit a prominent peak at 6007.1 eV, accompanied by two sub-peaks at 6009.8 and 6022.1 eV. The close agreement with standard Cr_2_O_3_ suggests that chromium is predominantly presented in the +3 oxidation state. Furthermore, Cr^4+^ ions generally exhibit a high degree of tetrahedral coordination stabilization, enabling the dipole-allowed transition of 1 s → 3d due to the mixing within the 3d and 4p states. However, the observed low intensity of the pre-edge feature at 5990.2 eV, associated with the 1 s → 3d transition, indicates that chromium selectively occupies the octahedral sites, where the 1 s → 3d transition is only quadrupole-allowed. Hence, the NIR-II emission is not related to Cr^4+^ impurities in tetrahedral sites. Additionally, the broad shoulder observed below 5980 eV can be attributed to the EXAFS signal originating from the La-L2 edge. Furthermore, the cryogenic (80 K) UV-Vis-NIR diffuse reflectance spectra show enhanced Cr^3+^ absorption, but no absorption signals of Cr^4+^ can be traced, as shown in Fig. 2d. Figures S7 and 2e comparatively illustrate the X-ray Photoelectron Spectroscopy (XPS) survey scan and high-resolution Cr 2p spectra, using Cr_2_O_3_ as the reference. The sharp line with a binding energy of 285 eV (C 1 s related) results from the adventitious hydrocarbons on the surface of the sample, while other peaks can be well indexed to core levels of constituent elements. As the concentration of Cr^3+^ increases to 0.7, the XPS profiles of Cr 2p doublets become almost consistent with the Cr_2_O_3_ reference without chemical shift, indicating no modification in the valence state of the host lattice. The dominance of NIR-II emission at higher Cr^3+^ concentration is generally associated with the Cr^3+^-Cr^3+^ aggregation (i.e. pairs and clusters). The EPR experiments (Fig. 2f) further suggest strong interaction among Cr^3+^ ions in the host lattice. The resonance signal at the low magnetic field region with g value of 4.25 is attributed to the isolated Cr^3+^ in the octahedral sites, while the resonance signal at g = 2.49 is ascribed to the large separation between the two Kramers’ doublets of |±3/2> and |±1/2>. The resonance signal at the high magnetic field region with g value of 1.96 is attributed to the coupling of Cr^3+^-Cr^3+^ pairs^41,42^. The broadening of this resonance profile at high field with increasing Cr^3+^ concentration indicates an enhanced interaction among Cr^3+^ ions.

In contrast to isolated dopants, the anomalous luminescence properties resulting from dopant aggregation are typically explained by magnetic interaction and IVCT. For Cr^3+^ in a strong crystal field environment, the first excited state is ^2^E, and the spin-forbidden emission (^2^E → ^4^A_2_) of the isolated center and Cr^3+^-Cr^3+^ magnetic coupling can be spectrally resolved due to these sharp-line features^43^. Besides, the spin selection rule can be relaxed, leading to a much shorter lifetime, as observed in LaAlO_3_:Cr^3+^^43,44^. However, LaMgGa_11_O_19_:Cr^3+^ exhibits broadband NIR emission, indicating a weak crystal field environment of Cr^3+^. Energy values *E(S)* associated with the spin interaction between two activators A and B can be approximately determined by the following equation^43^:

$$E(S)=-J[S(S+1)-{S}_{A}({S}_{A}+1)-{S}_{B}({S}_{B}+1)]$$

where *J* is the spin coupling strength parameter, *S*_*A*_ and *S*_*B*_ are the spin quantum numbers of activators A and B, and *S* is the total spin quantum number. Therefore, the value of *E(S)* significantly depends on *S* and *J*. In a weak crystal field environment, the ground and first excited states of Cr^3+^-Cr^3+^ magnetic coupling are [^4^A_2_(F),^4^A_2_(F)] and [^4^A_2_(F),^4^T_2_(F)]. Thus, the total spin quantum number *S* can be obtained through (*S*_*A*_ + *S*_*B*_), …, (*S*_*A*_-*S*_*B*_), i.e. S = 3, 2, 1, 0. Considering that the coupling parameter *J* is generally within tens of wavenumbers^43^, the maximum *E(S)* is approximately 450 cm^−1^. Similarly, the maximum value of *E*(*S*) under a strong crystal field environment is approximately 150 cm^-1^. However, for the 0.7Cr^3+^ sample, the energy difference between the maximum of NIR-I and NIR-II emissions is 3715 cm^−1^. Therefore, the NIR-II luminescence cannot be attributed to the magnetic interaction of Cr^3+^-Cr^3+^ pair. In fact, the ^4^T_2_ → ^4^A_2_ broadband emission of isolated Cr^3+^ and [^4^A_2_(F),^4^T_2_(F)] → [^4^A_2_(F),^4^A_2_(F)] of magnetic interaction cannot be spectrally resolved due to the small value of *E(S)*, which results in a significant spectral overlap.

### Luminescence decay lifetimes

**Fig. 3:**
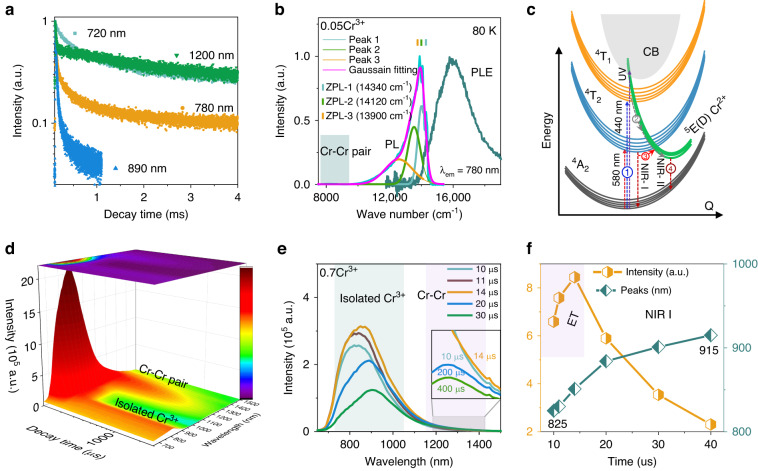
Luminescence decay and schematic IVCT process. **a** Cryogenic (80 K) luminescence decay curves of LaMgGa_11_O_19_:0.05Cr^3+^ sample monitoring at 720, 780, 890, and 1200 nm. **b** Magnified cryogenic PL and PLE spectra of LaMgGa_11_O_19_:0.05Cr^3+^ sample to estimate the ZPL energies. **c** Schematic luminescence mechanism based on IVCT. **d** Luminescence decay 3D color mapping of LaMgGa_11_O_19_:0.7Cr^3+^ sample. **e** Time-resolved PL spectra from 10–30 μs. The inset shows the magnified patterns ranging from 1180 to 1420 nm within 10–400 μs. **f** Scatters of luminescence intensity and peaks versus decay time

The metal-to-metal IVCT state is advantageous in regulating absorption, emission, and non-radiative transition because it is intertwined among the configuration levels of isolated optical-active centers. As Cr^3+^-Cr^3+^ aggregation increases, electron transfer from one Cr^3+^ ion to its neighbor becomes possible, resulting in the formation of Cr^4+^ and Cr^2+^ pair, e.g. Cr^3+^-Cr^3+^ → Cr^2+^-Cr^4+^. If radiative recombination follows, the spontaneous emission typically exhibits a large Stokes-shift, as seen in the PL spectrum in Fig. S5. Luminescence decay curves further support this assumption, as demonstrated in Fig. 3a, which comparatively presents luminescence lifetime decay curves at 80 K. All the decay curves can be well fitted by bi-exponential functions^45^:

1 $$I={A}_{1}\exp \left(\frac{t}{{\tau }_{1}}\right)+{A}_{2}\exp \left(\frac{t}{{\tau }_{2}}\right)$$

where *I* denotes the luminescence intensity, *τ*_1_ and *τ*_2_ refer to the decay lifetimes of the first and second exponential fitting, *A*_1_ and *A*_2_ are the exponential fitting constants of *τ*_1_ and *τ*_2_. In this case, the average lifetime values can be obtained using the following equation:

2 $${\tau }_{average}=\frac{{A}_{1}{{\tau }_{1}}^{2}+{A}_{2}{{\tau }_{2}}^{2}}{{A}_{1}{\tau }_{1}+{A}_{2}{\tau }_{2}}={f}_{1}{\tau }_{1}+{f}_{2}{\tau }_{2}$$

where *f*_1_ and *f*_2_ denote the percentages of *τ*_1_ and *τ*_2_ components. Bi-exponential fitting results are presented in Fig. S8. Accordingly, the average decay lifetime monitoring at 720 nm is 1.47 ms, attributed to the spin-forbidden ^2^E → ^4^A_2_ transition and associated overtones. Monitoring at 780 nm broadband emission, the decay lifetime of 541 μs is longer than that of other Cr^3+^ doped compounds in the weak crystal field environment. We attribute this to the strong coupling between the ^2^E and ^4^T_2_ configurations. Figure 3b presents the PLE and PL spectra to determine the zero-phonon line (ZPL) energies, which are determined by the intersection of PLE and PL spectra. The energy gap between the sharp R-line (698 nm) and ZPL is only within a few hundred wavenumbers, indicating a strong coupling between the ^2^E and ^4^T_2_ configurations. Monitoring at 890 nm, the lifetime is only 89 μs, indicating a weak crystal field environment. In summary, the luminescence decay curves monitoring at NIR-I emission indicate multiple isolated Cr^3+^ centers with different crystal field environment, consistent with the PL spectra. However, the luminescence lifetime monitoring at 1200 nm is 2.3 ms, much longer than that monitoring at NIR-I emission. Moreover, this decay lifetime is significantly longer than typical Cr^4+^, which is only a few tens of microseconds^46,47^. This further confirms that the NIR-II emission is unrelated to Cr^4+^ impurity. In general, the spontaneous radiation of the Cr^3+^ *d*-*d* parity-forbidden transition is relaxed due to the mixing in the 3*d*^3^ state with other opposite parity. Thus, the emission is partially electric dipole allowed. The spontaneous radiative lifetime *τ*_IF_ from the initial state *I* to final excited state *F* versus radiative wavelength *λ*_IF_ can be written as^48^:

3 $${\varGamma }_{IF}=\frac{1}{{\tau }_{IF}}=\frac{64{\pi }^{4}}{3h{{\lambda }_{IF}}^{3}}\chi {|\overrightarrow{{\mu }_{IF}}|}^{2}$$

where *Γ*_IF_ denotes the spontaneous radiative rate, *h* is Planck’s constant, and ${\overrightarrow{\mu }}_{{\rm{IF}}}$ is the dipole moment between *I* and *F*. Therefore, the decay lifetime *τ* is proportional to *λ*^3^. After emission wavelength correction, the luminescence lifetimes monitoring at NIR-II emission are still longer than those monitoring at NIR-I emission, as listed in Table S2. For magnetic interaction, luminescence lifetimes generally shorten in Cr^3+^ and Mn^2+^ doped compounds, depending greatly on the exchange-coupling parameter *J*^43,44^. However, for the IVCT pair emission, luminescence decay lifetime is longer than that of the isolated activator^49,50^. The IVCT transition of Cr^3+^-Cr^3+^ → Cr^2+^-Cr^4+^ occurs with a configurational transition between two neighboring Cr^3+^, potentially with low absorption probability compared to the ^4^T_2_ → ^4^A_2_ inter-configurational transition. Accordingly, the luminescence lifetime is longer. Furthermore, isolated Cr^3+^ exhibits multiple excited-state quenching process, for example energy transfer among Cr^3+^, re-absorption. However, the IVCT luminescence exhibits much larger stokes-shift, with no overlap observed between PL and PLE spectra. Accordingly, the energy transfer and re-absorption process are effectively inhibited, which also possibly leads to the longer luminescence lifetime. Thus, the anomalous NIR-II emission with a large Stokes-shift originates from the IVCT transition (Cr^3+^-Cr^3+^ → Cr^2+^-Cr^4+^) rather than the magnetic interaction. However, the direct excitation signal of the IVCT cannot be discerned from the UV-vis-NIR diffuse reflection and PLE spectra. Our speculation is that the IVCT transition of Cr^3+^-Cr^3+^→ Cr^2+^-Cr^4+^ involves a charge transfer transition between two neighboring Cr^3+^ centers, resulting in a lower absorption probability compared to the stronger ^4^A_2_ → ^4^T_2_ intra-configurational transition of isolated Cr^3+^ ions. Consequently, the excitation intensity of the IVCT band is significantly reduced. Furthermore, the IVCT excitation is typically located in the UV region or even at higher energy levels, making it challenging to differentiate due to its overlap with the excitation transitions of ^4^A_2_ → ^4^T_2_(F), ^4^A_2_ → ^4^T_1_(P), the O^2-^→Cr^3+^ charge transfer, interband absorption, etc. Furthermore, despite the intervalence charge transfer of Cr^3+^-Cr^3+^ → Cr^2+^-Cr^4+^ leads to the partial oxidation and reduction of Cr^3+^. However, the charge transfer transition happens instantaneously, and the XANES, XPS, EPR, and PLE characterizations are steady-state test methods at a macroscopic level. Consequently, the signal associated with Cr^4+^/Cr^2+^ are challenging to be observed.

Figure 3c constructs a configuration coordinate diagram to explain the IVCT-based NIR-II luminescent mechanism. Firstly, the electron is photo-pumped to the excited states of isolated Cr^3+^ under UV-NIR excitation (①), followed by de-excitation to ^4^T_2_(F) state (②) to produce the NIR-I emission. The IVCT excited state can be understood as the result of the electron transfer between a pair of Cr^3+^ ions, leading to the formation of the product of two ground states involving Cr^2+^ and Cr^4+^ ions (②). In simpler terms, the transition occurring between these two centers can be broken down into two separate single-center processes: the oxidation of a Cr^3+^ ion and the reduction of another Cr^3+^ ion. Following the standard practice on the formation energies of different charge states^51^, we can determine the energy position of the ground ^4^A_2_ state of the Cr^3+^ ion relative to the top of the valence band by considering the energy change before and after the oxidation reaction Cr^3+^ → e(VB) + Cr^4+^, where e(VB) represents an electron located on the top of the valence band. Similarly, we can define the energy position of the ground state ^5^E of the Cr^2+^ ion for description of the reduction reaction Cr^3+^ + e(VB) → Cr^2+^. Therefore, the energy difference between the ground states of the Cr^3+^ and Cr^2+^ ions provides a clear estimation of the IVCT energy, indicating that the related IVCT emission undoubtedly occurs between these two states rather than involving the ground state of the Cr^4+^ ion. It is clear that the condition for the occurrence of IVCT emission is that the ^5^E ground state of the Cr^2+^ ion is situated below the first excited state ^4^T_2_ of the Cr^3+^ ion. The energy position of the ^4^T_2_ state with respect to the top of the valence band can be evaluated by adding the energy position of the ground ^4^A_2_ state of the Cr^3+^ ion to the transition energy from the ^4^A_2_ to ^4^T_2_ states of the Cr^3+^ ion. A similar transition process can be observed in the case of a pair of Bi^2+^ and Bi^4+^ ions, leading to two Bi^3+^ ions, as depicted in Fig. 2 of the reference^49^. However, due to the lack of spectral data on Cr^2+^, it is challenging to determine the energy level of Cr^2+^ in the LaMgGa_11_O_19_ host.

Time-resolved emissions (TRES) color mapping is further shown in Fig. 3d to identify the ET process among multiple Cr^3+^ centers. With increasing decay time, the emission intensity at 890 nm first increases and then quenches. Additionally, the emission peaks continuously shift toward longer wavelengths (Figs. 3e and f), indicating an efficient ET process among multiple Cr^3+^ centers. In addition to the NIR-I emission, the NIR-II emission also exhibits an efficient ET process due to the initial increase in luminescence intensity, as demonstrated in Fig. 3e and S9.

### Luminescence thermal stability and efficiency

**Fig. 4:**
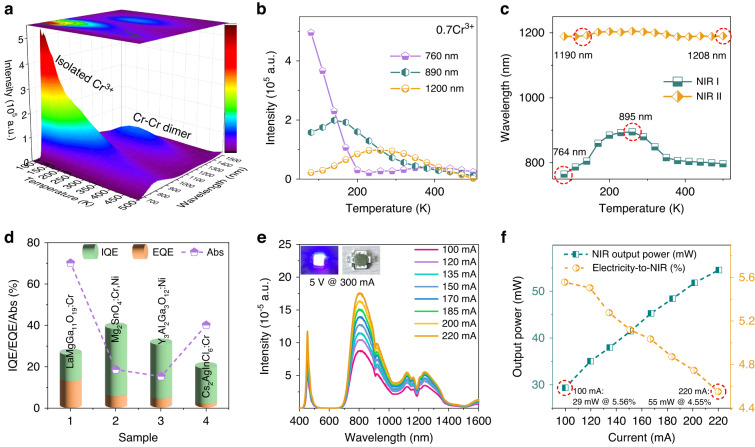
Luminescence thermal stability and photoelectric properties of NIR pc-LED. **a** Temperature-dependent luminescence 3D color mapping of LaMgGa_11_O_19_:0.7Cr^3+^ sample. **b** Luminescence intensities versus temperature monitoring at 760, 890, and 1200 nm. **c** Scatters of peak positions versus temperature. **d** Comparison of IQE, EQE, and Abs values with some reported NIR-II luminescent phosphor including Mg_2_SnO_4_:Cr^3+^,Ni^2+^, Y_2_Al_2_Ga_3_O_12_:Ni^2+^, and Cs_2_AgInCl_6_:Cr^3+^. **e** Electroluminescence spectra and photographs of the fabricated NIR pc-LED device by integrating LaMgGa_11_O_19_:0.7Cr^3+^ on a commercial 450 nm blue LED. **f** NIR output power and photoelectric conversion efficiency of the fabricated pc-LED with the tunable driven current

Figure 4a illustrates temperature-dependent luminescence 3D color mapping. The integrated intensity decreases continuously with increasing temperature from 80 to 500 K, due to the enhancement of nonradiative transition probabilities, as shown in Fig. S10. At 500 K, the integrated intensity is only ~15.6% of that at 80 K. However, the emission intensities monitored at 890 and 1200 nm exhibit anti-thermal quenching behavior, which means that they first increase and then quench above 140 and 290 K, respectively, as depicted in Fig. 4b. Notably, the luminescence intensity of 1200 nm is approximately 4.3 times higher than that at 80 K. In addition, the NIR-I emission displays a significant red-shift from 764 to 895 nm (Fig. 4c), indicating an efficient ET process among multiple Cr^3+^ centers. The structural analysis reveals that the Ga4 site has the longest Ga-O bond length (Table S3), which is responsible for the 890 nm broadband emission. Moreoever, Ga4-O octahedra are connected by face-sharing with the shortest Ga-Ga distance, enabling 1200 nm luminescence of Cr^3+^-Cr^3+^ → Cr^2+^-Cr^4+^. As the temperature increases, ET from Ga1/Ga5 to Ga4 site intensifies, leading to the experimentally observed anti-thermal quenching behavior. Additionally, with thermal assistance (③, Fig. 3c), partial electrons selectively transfer from ^4^T_2_(F) to the IVCT state. As a result, the luminescence intensity monitored at 890 nm decreases above 140 K, whereas that at 1200 nm continues to increase by 4.32 times until 290 K.

Reporting of the luminescence internal/external quantum efficiency (IQE/EQE) in the NIR-II region has been scare due to the limited experimental setup. Cr^4+^ and Ni^2+^ are considered as ideal NIR-II broadband light-emitting centers, but they exhibit low luminescence efficiency, for instance, 9.8% EQE for NIR-I to NIR-II emission of Mg_2_SnO_4_:Ni^2+^^52^, and 8.2% EQE for NIR-II emission of Y_3_Al_2_Ga_3_O_12_:Ni^2+^^53^. On the other hand, for the 0.7 Cr^3+^ sample, the IQE of NIR luminescence is estimated to be 27.2% under 440 nm excitation, as illustrated in Fig. 4d. Due to the high absorption rate (69.7%) resulting from the heavy Cr^3+^ incorporation, the EQE is up to 18.9% higher than the above mentioned values. However, the IQE and EQE values of anomalous NIR-II luminescence are only 14.0% and 9.7%.

### LED fabrications and applications

To enable practical applications in photo-converted broadband NIR light source, LaMgGa_11_O_19_:0.7Cr^3+^ dual-emitting phosphor was integrated onto a commercially available blue-emitting chip (450 nm) to fabricate the NIR pc-LED device. Figure 4e shows the electroluminescence spectra with tunable driven current from 100 to 220 mA. The electroluminescence spectra are different from the PL spectra. This is ascribed to the different optical detector, i.e. charge coupled device (CCD) for electroluminescence spectra while photomultiplier tube (PMT) for PL spectra. As the driven current increases, the luminescence intensity continuously grows, and the NIR output power increases from 29 to 55 mW (Fig. 4f). However, the electricity-to-NIR conversion efficiency continuously decreases due to the “efficiency droop” of the blue-emitting chip and the luminescence thermal quenching of phosphor. Notably, the NIR output power and electricity-to-NIR conversion efficiency under 100 mA driven current are 29 mW and 5.56%, respectively.

**Fig. 5:**
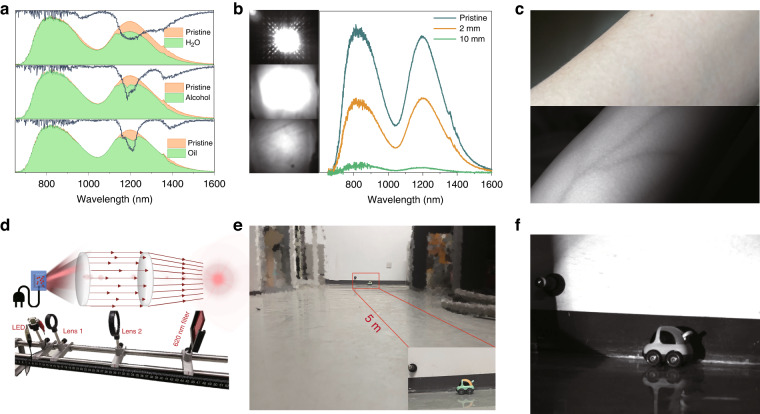
Multiple applications using NIR pc-LED. **a** PL spectra and absorption spectra after penetrating the water, absolute alcohol, and oil. **b** NIR luminescence images and spectra of the fabricated pc-LED device after penetrating the 2 and 10 mm beef tissue. **c** The photography of the human arm under indoor lighting and NIR pc-LED. **d** The schematic diagram of beam homogenizer and condenser systems for long-distance night vision. **e** The photography of the car with an ordinary camera under indoor lighting at a distance of 5 meters. **f** The photography of the car with an NIR camera under the fabricated pc-LED

Broadband NIR spectroscopy provides massive information about the molecular structures and chemical compositions of organic compounds, making it a favorable method for non-destructive analysis. Figure 5a displays the luminescence spectra before and after penetrating water, absolute alcohol, and oil using the dual-emitting LaMgGa_11_O_19_:Cr^3+^ phosphor. The luminescence attenuations after penetrating these substances are quite different due to their distinct absorption responses toward the NIR region. Alcohol and oil exhibit significant absorptions around 1200 and 1400 nm, which are attributed to the second overtone of the C-H stretching vibration and the first overtone of the O-H stretching vibration. In contrast, water exhibits absorption around 980, 1200, and 1370 nm, which are attributed to the characteristic overtones of O-H stretch. These absorption responses indicate the potential application of this broadband dual-emitting phosphor in non-destructive analysis of agriculture.

Moreover, the NIR-II region features low tissue absorption and scattering coefficients, resulting in a large probing depth and low autofluorescence. Tissue-penetration experiments were further implemented using this NIR pc-LED as a light source, and a 645 nm filter was used to screen out the blue light. Figure 5b illustrates the luminescence spectra and brightness images after penetrating beef tissue. Although the luminescence intensity continuously decreases with increasing tissue thickness, a bright image can be perceived after penetrating 10 mm of tissue. In comparison with living tissue, blood and arteries exhibit much larger absorption coefficients to NIR radiation, enabling efficient vessel localization for infusion application. Additionally, compared with the widely reported angiography based on NIR light-emitting phosphors penetrating living tissue (e.g. human palm), angiography based on light reflection from the tissue surface requires less input power and light loss. Figure 5c depicts the conceptual angiography experiment using this NIR pc-LED as a light source based on light reflection. The result indicates that the black-and-white image of the human arm can be clearly perceived, revealing the internal vascular distribution through shadow dendrites. In contrast, it’s difficult to distinguish the artery under indoor lighting.

Furthermore, the naked eyes exhibit low sensitivity to NIR radiation, enabling the use of NIR-emitting phosphors for night vision applications. However, compact NIR pc-LEDs generally exhibit low light output and large light divergence, making them incapable of long-distance night vision and tracing. Therefore, easy-handled beam homogenizer and condenser systems containing two convex lenses were constructed to obtain convergent NIR light, as schematically shown in Fig. 5d. The convergence distance can be effectively adjusted by altering the distance between the LED and the lens 1. Figure 5e displays the photography of a car with an ordinary camera under indoor lighting at a distance of 5 meters. The object can be directly captured but not in the dark. In contrast, with the aid of the fabricated NIR light-emitting devices, a clear black-and-white photography of the car can be captured under the NIR camera, as shown in Fig. 5f.

## Conclusion

In conclusion, this study reports the first-ever broadband NIR-II luminescence based on Cr^3+^-Cr^3+^ aggregation in magentoplumbite-type LaMgGa_11_O_19_. Heavy Cr^3+^ incorporation results in dual-emitting NIR luminescence (890 and 1200 nm) with an FWHM of 626 nm and EQE of 18.9% upon 440 nm excitation. Moreover, this dual-emission exhibits anti-thermal quenching behavior (432% @ 290 K), attributed to the energy transfer among multiple Cr^3+^ centers. Cryogenic diffuse reflectance, XPS, EPR, XANES, and luminescence lifetimes validate that the NIR-II emission is unrelated to Cr^4+^ impurity and magnetic exchange coupling but is due to the IVCT of Cr^3+^-Cr^3+^ → Cr^2+^-Cr^4+^ between neighboring Cr^3+^-Cr^3+^ pairs. Furthermore, the potential application in non-destructive analysis, tissue penetration, and long-distance night vision using LaMgGa_11_O_19_:Cr^3+^ as a photo-converted NIR light-emitting source is demonstrated. This work provides new insights into Cr^3+^ luminescence and opens up new strategies for efficient broadband NIR-II luminescent phosphors based on the Cr^3+^-Cr^3+^ aggregation.

## Supplementary information

Supplementary Information for Intervalence charge transfer of Cr^3+^-Cr^3+^ aggregation for NIR-II luminescence
