# Supplementary material for: Intervalence charge transfer of Cr3+-Cr3+ aggregation for NIR-II luminescence
Source: Light Sci Appl. 2023 Jul 25;12:181. doi: 10.1038/s41377-023-01219-x (PMC10366090; doi:10.1038/s41377-023-01219-x)
Supplement: Supplementary file 1 — Supplementary Information for Intervalence charge transfer of Cr3+-Cr3+ aggregation for NIR-II luminescence [file 41377_2023_1219_MOESM1_ESM.docx]

**Supplementary Information for**

**Intervalence charge transfer of Cr^3+^-Cr^3+^ aggregation for NIR-Ⅱ luminescence**

Shengqiang Liu,^1^ Jingxuan Du,^1^ Zhen Song,^1*^ Chonggeng Ma,^2*^ Quanlin Liu.^1*^

^1^ Beijing Municipal Key Laboratory of New Energy Materials and Technologies, School of Materials Sciences and Engineering, University of Science and Technology Beijing, Beijing 100083, China.

E-mail: zsong@ustb.edu.cn (Z. Song)

E-mail: qlliu@ustb.edu.cn (Q. Liu)

^2^ School of Optoelectronic Engineering & CQUPT-BUL Innovation Institute, Chongqing University of Posts and Telecommunications, Chongqing 400065, China.

E-mail: cgma.ustc@gmail.com (C. Ma)

**Experimental Section**

**Samples synthesis**

All the NIR phosphors were synthesized using a high-temperature solid reaction method. Starting reagents including La_2_O_3_ (99.99%), SrCO_3_ (99.9%), MgO (99.99%), Ga_2_O_3_ (99.99%), and Cr_2_O_3_ (99.9%) were weighted stoichiometrically with 1wt% H_3_BO_3_ as flux. These raw powders were thoroughly ground for 30 min with ethanol in an agate mortar and then transferred to an alumina crucible. Samples were sintered at 1450 ^o^C for 6 h. After cooling to room temperature naturally, the as-synthesized samples were ground into fine powders and packed for subsequent characterizations.

**Characterizations**

X-ray diffraction (XRD) patterns were collected on a diffractometer (TTR Ⅲ, Rigaku, Japan) with a Cu Kα radiation source operating at 40 kV and 200 mA. The Rietveld structural refinement was conducted using the Fullprof program with 2θ from 5-120 degrees and the Inorganic Crystal Structure Database structure model #JCPDS84-0889 was used as the starting model for phase determination. The bond valence sum (BVS) was calculated using the Bond-Str program. X-ray Photoelectron Spectroscopy (XPS) was measured on a spectrometer (Thermo SCIENTIFIC ESCALAB 250Xi) with an Al Kα radiation source. The electron paramagnetic resonance (EPR) was measured on a EMX-10/12 spectrometer (Bruker Ltd.) with 20 mW output. The particle morphology and elemental mappings were collected by scanning electron microscopy (SEM, JEOL JSM-6510A). The photoluminescence (PL), photoluminescence excitation (PLE) and luminescence quantum efficiency were recorded on an FLS1000 spectrophotometer (Edinburgh Instrument Ltd., U.K) equipped with a 500 W Xe lamp. The luminescence decay curves were recorded on the FLS1000 using μF900 flash lamp as pump light source and the lifetime fitting was conducted using the FAST program integrated into the FLS1000. The temperature-dependent PL spectra were also recorded on the FLS1000 using a MercuryiTc temperature control instrument (OXFORD, U.K), and liquid nitrogen was used as the cooling source. The room temperature and cryogen diffuse reflectance curves were recorded on UH4150 (Hitachi) and UV-3600 (Shimadzu) UV-Vis-NIR spectrophotometer, with Polytetrafluoroethylene (PTFE) as the calibration. The photoelectric properties of the fabricated NIR pc-LED were recorded using a HAAS2000 photoelectric measuring system (EVERFINE, China).


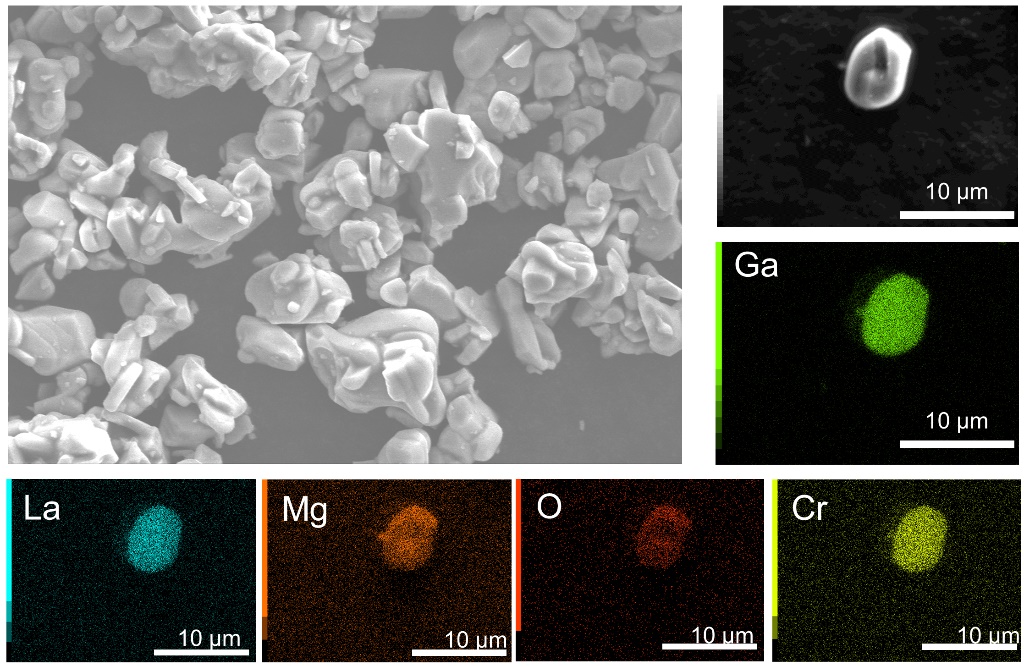


Figure 1. SEM microscopic morphology image and elemental mappings of LaMgGa_10.3_O_19_:0.7Cr^3+^ sample.


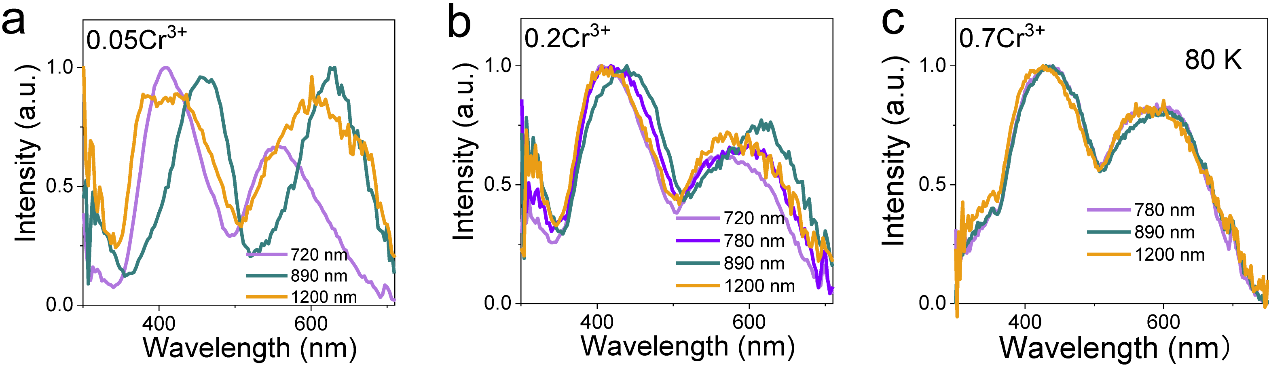


Figure S2. Cryogen (80 K) PLE spectra of a) 0.05 Cr^3+^, b) 0.2 Cr^3+^, and c) 0.7 Cr^3+^ monitoring at different emission wavelengths.


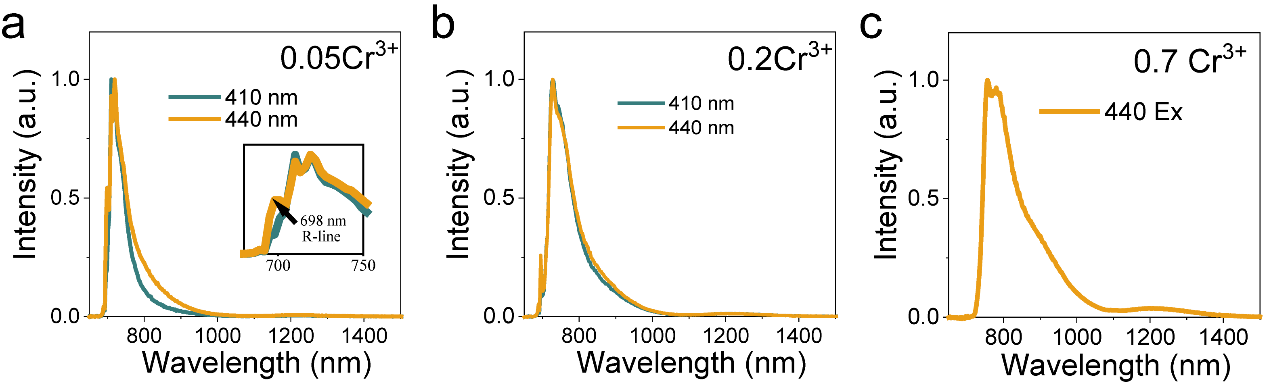


Figure S3. Cryogen (80 K) PL spectra of a) 0.05 Cr^3+^, b) 0.2 Cr^3+^, and c) 0.7 Cr^3+^ excited by 410 and 440 nm. The inset in a) shows the magnifying patterns from 675 to 750 nm.


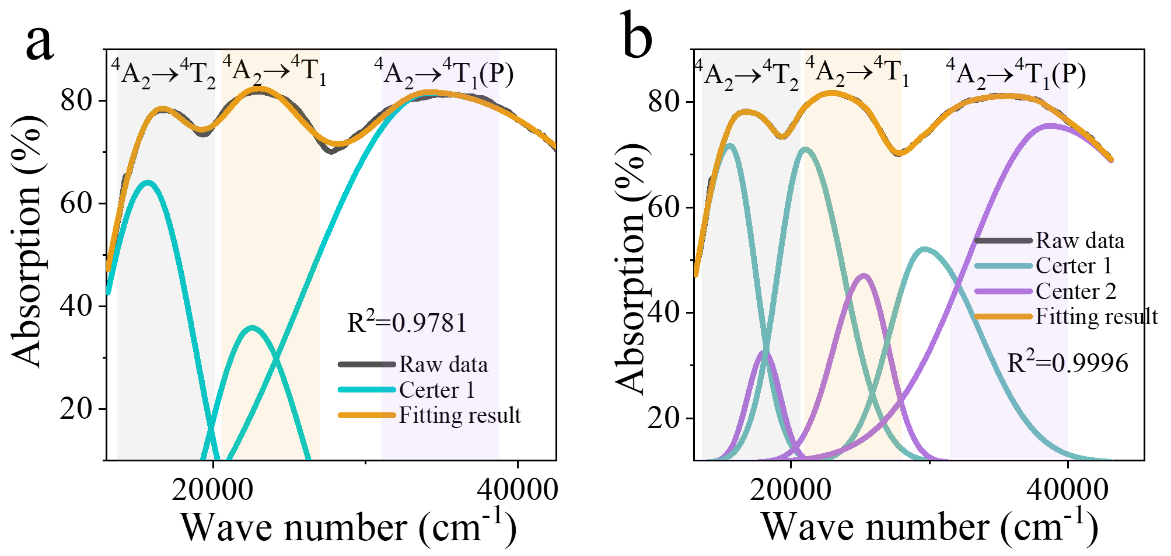


Figure S4. Bi-Gaussian deconvolution of cryogen (80 K) absorption curves by a) a single Cr^3+^ center and b) two Cr^3+^ centers.

**Identification of multiple Cr^3+^ centers**

To explore the actual Cr^3+^ centers in the LaMgGa_11_O_19_ host, Figure S2 comparatively shows the cryogen PLE spectra (80 K) monitoring at different emissions. Doping only 0.05 Cr^3+^, the PLE spectrum monitoring at 890 nm shows a large red-shift compared with that at 720 nm, indicating multiple isolated Cr^3+^ centers in the different crystal field environments. In hexagonal LaMgGa_11_O_19_ host, Ga1 2*a*, Ga4 4*f*, and Ga5 12*k* are six-oxygen coordinated, capable for Cr^3+^ incorporation. Especially, the Ga1 site shares the shortest Ga-O bond length, while the longest Ga-O bond length for the Ga4 site, as listed in Table S3. Consequently, the sharp R-line (698 nm) and associated overtones at 80 K in Figure S3a are ascribed to isolated Cr^3+^ in the Ga1 site with the strongest crystal field environment, and the isolated Cr^3+^ in Ga4 and Ga5 sites dominate the broadband NIR-Ⅰ emission (650-1000 nm). With increasing Cr^3+^ concentration, the PLE spectra monitoring at 890 nm show continuous blue-shift and finally coincide with the excitation spectra monitoring at 720 nm. This indicates that partial excitation monitoring at 890 nm originates from the Ga1 crystallographic site, due to enhanced ET from Ga1 to Ga4/Ga5 sites. For the 1200 nm emission, the PLE spectrum of 0.05Cr^3+^ sample is much broader and nearly covers the excitation spectral range of that monitoring at 720 and 890 nm. With increasing Cr^3+^ concentration, the PLE spectra gradually converge to the same profiles. Absorption features can exclude the effect of ET among multiple luminescence centers. Figure S4 shows the bi-gaussian functions fitting of cryogen UV-Vis-NIR diffuse reflectance spectrum of 0.7 Cr^3+^ sample. The result shows that the absorption profile of ^4^A_2_→^4^T_1_ and ^4^A_2_→^4^T_2_ transition cannot be well fitted by a single Cr^3+^ center, but at least two Cr^3+^ centers in the LaMgGa_11_O_19_ matrix.


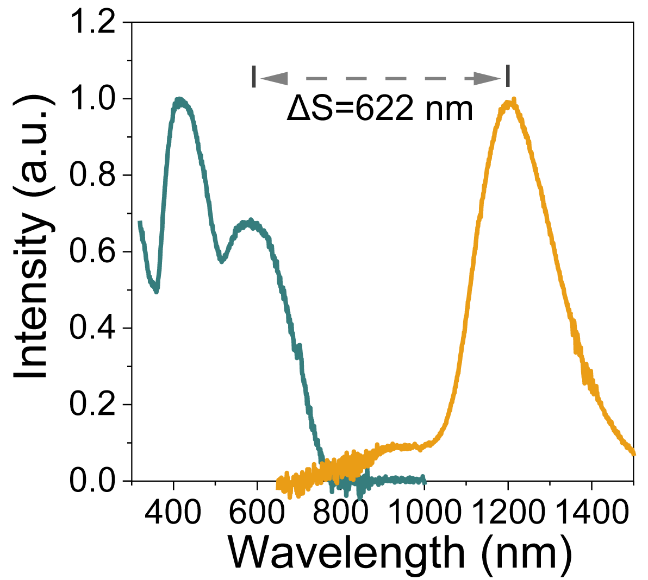


Figure S5. PL and PLE spectra of LaMgGa_10_O_19_:1Cr^3+^.


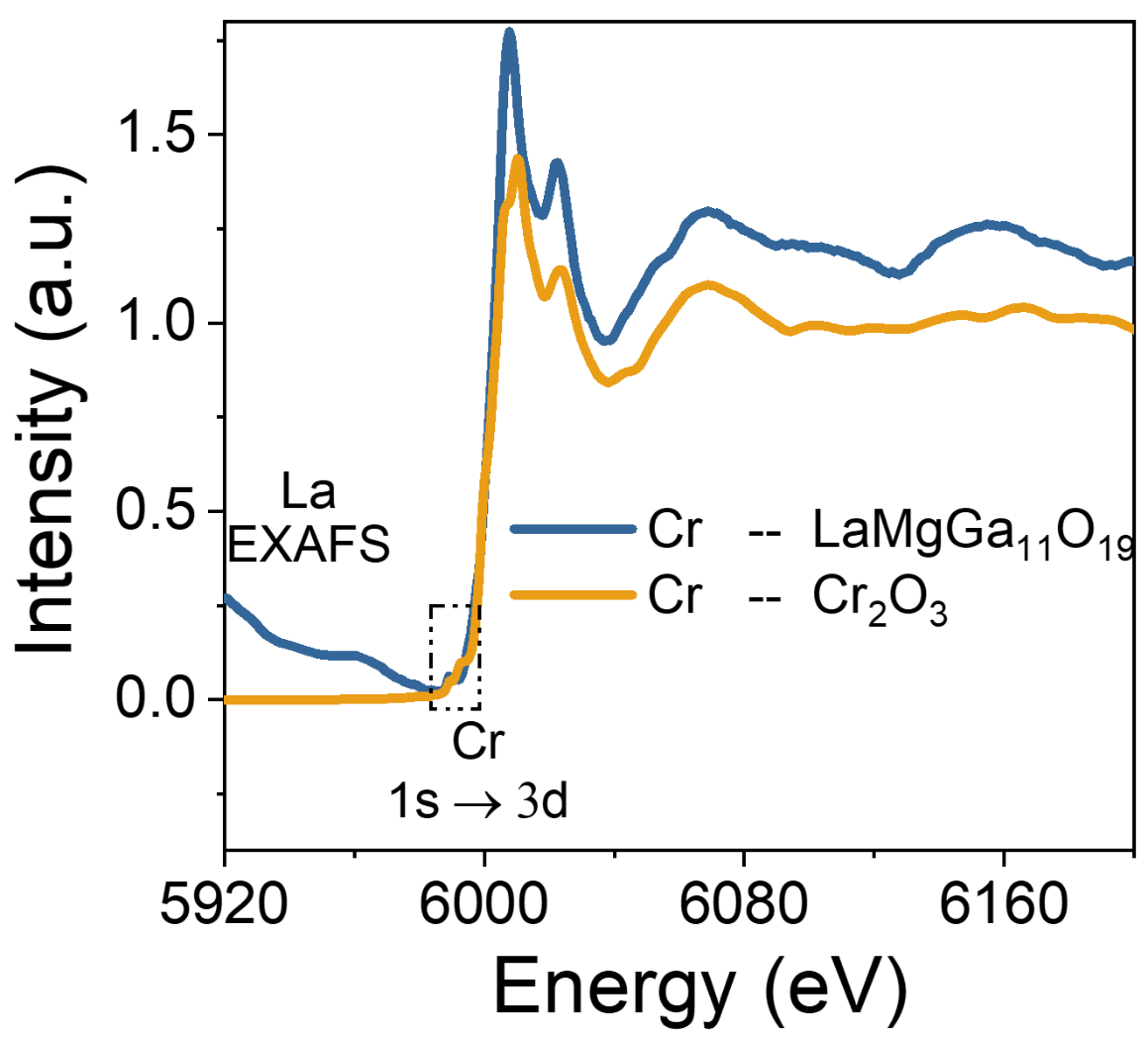


Figure S6. Cr-K edge XANES spectra of LaMgGa_11_O_19_:0.7Cr^3+^ and Cr_2_O_3_.


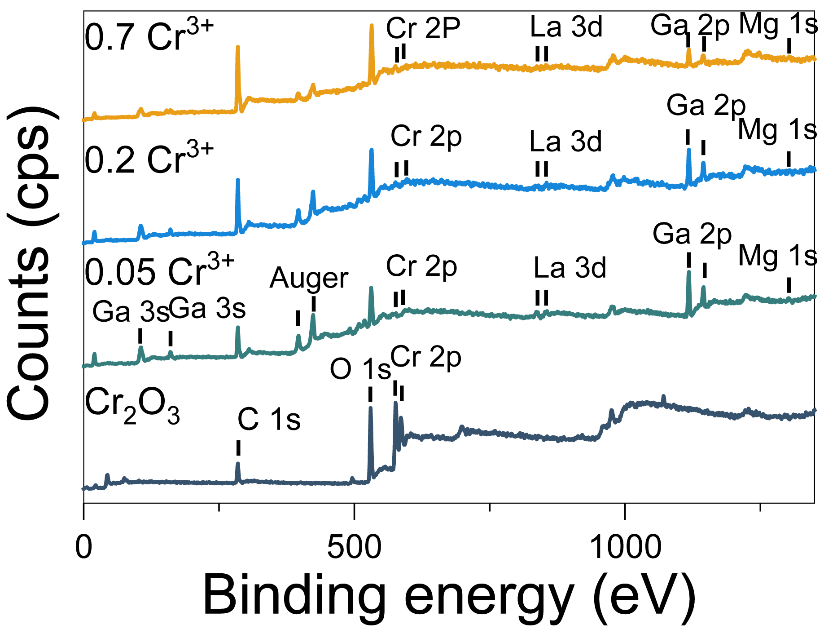


Figure S7. XPS survey scan of LaMgGa_11-_*_x_*O_19_:*x*Cr^3+^ (*x*=0.05, 0.2, 0.7) samples with Cr_2_O_3_ used as the reference.


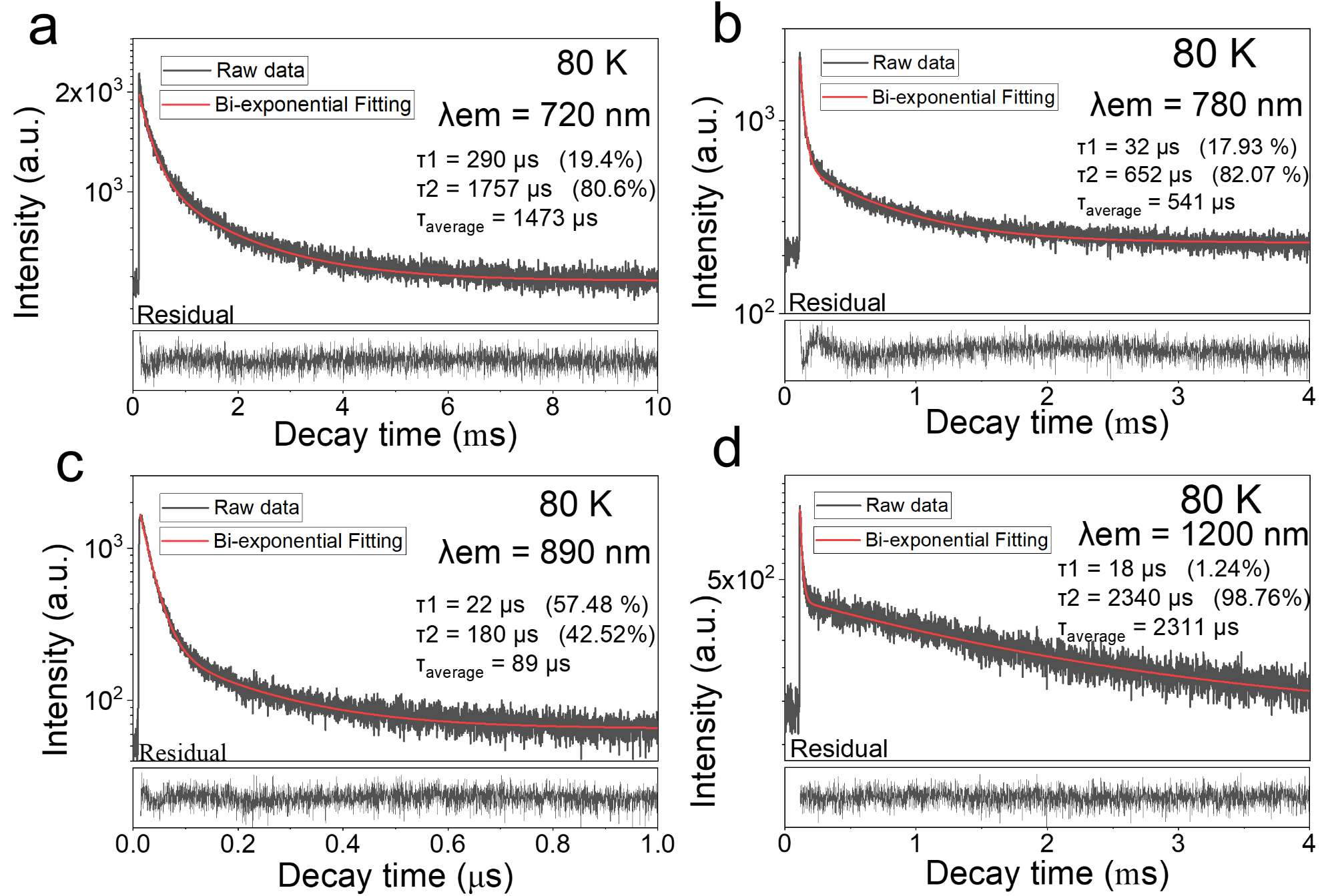


Figure S8. Cryogen (80 K) luminescence decay curves and bi-exponential fitting results of 0.2 Cr^3+^ sample monitoring at a) 720 nm, b) 780 nm, c) 890 nm, and d) 1200 nm.


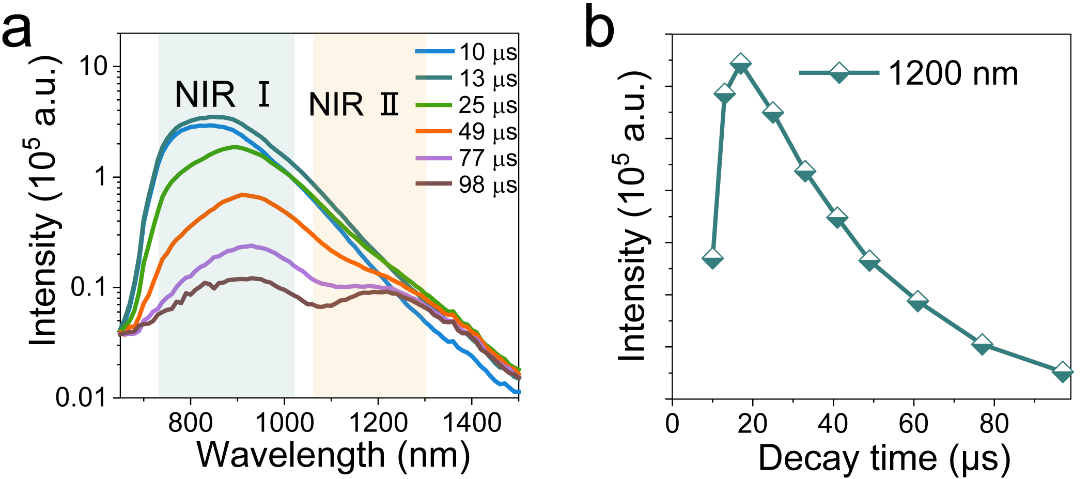


Figure S9. a) Time-resolved emissions spectra (10-98 μs) of 0.7 Cr^3+^ sample. b) Luminescence intensity monitoring at 1200 nm versus decay time scatters.


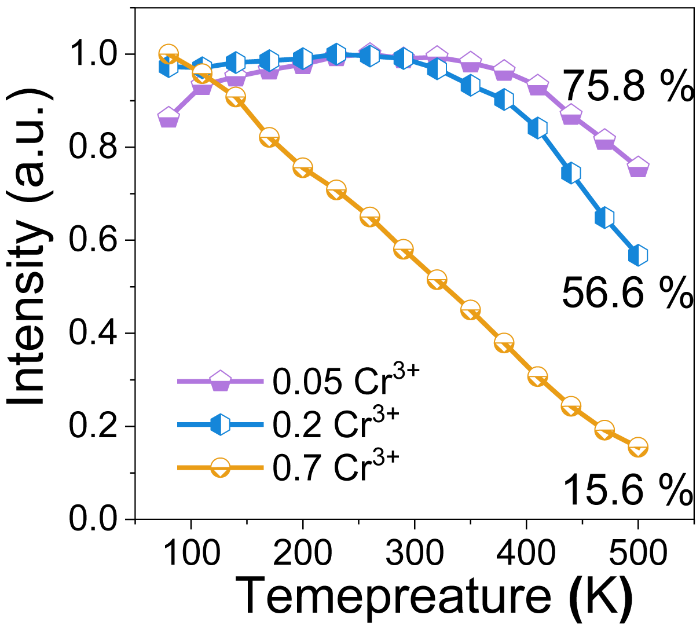


Figure S10. Temperature-dependent luminescence integrated intensity scatters of 0.05, 0.2, and 0.7 samples.

Table S1. The refined structural parameters of LaMgGa_10_O_19_:1Cr^3+^.

|  | Atom | x | y | z | Occ. | Site |  |  |
| --- | --- | --- | --- | --- | --- | --- | --- | --- |
| 1 | Ga^1^ | 0.000 | 0.000 | 0.000 | 0.700 | 2*a* | space group | *P 6_3_/mmc* (194) |
| 2 | Mg^1^ | 0.000 | 0.000 | 0.000 | 0.250 | 2*a* |  |  |
| 3 | Cr1 | 0.000 | 0.000 | 0.000 | 0.050 | 2*a* |  |  |
| 4 | Ga^2^ | 0.000 | 0.000 | 0.241(3) | 0.500 | 4*e* |  |  |
| 5 | Ga^3^ | 0.333 | 0.666 | 0.026(8) | 0.625 | 4*f* | symmetry | hexagonal |
| 6 | Mg^3^ | 0.333 | 0.666 | 0.026(8) | 0.375 | 4*f* |  |  |
| 7 | Ga^4^ | 0.333 | 0.666 | 0.188(4) | 0.800 | 4*f* | Lattice parameters (Å) | *a*=5.805 |
| 8 | Cr4 | 0.333 | 0.666 | 0.188(4) | 0.200 | 4*f* |  |  |
| 9 | Ga^5^ | 0.833(3) | 0.166(7) | 0.108(3) | 0.890 | 12*k* |  | *c*=22.702 |
| 10 | Cr5 | 0.833(3) | 0.166(7) | 0.108(3) | 0.110 | 12*k* |  |  |
| 11 | O^1^ | 0.000 | 0.000 | 0.150(3) | 1.000 | 4*e* |  |  |
| 12 | O^2^ | 0.666 | 0.333 | 0.057(9) | 1.000 | 4*f* | *R_p_* | 8.115 |
| 13 | O^3^ | 0.182(0) | -0.182(0) | 0.250 | 1.000 | 6*h* | *R_w_* | 10.243 |
| 14 | O^4^ | 0.152(0) | -0.152(0) | 0.053(3) | 1.000 | 12*k* | *R_exp_* | 5.117 |
| 15 | O^5^ | 0.505(0) | -0.505(0) | 0.151(3) | 1.000 | 12*k* |  |  |
| 16 | La^1^ | 0.666 | 0.333 | 0.250 | 0.770 | 2*d* |  |  |
| 17 | La^2^ | 0.732(3) | 0.267(7) | 0.250 | 0.076 | 6*h* |  |  |

The relationship of spontaneous radiative lifetime *τ*_IF_ from initial state *I* to final excited state *F* and radiative wavelength λ_IF_ can be described by following: ^[1,2]^

$$\Gamma_{IF}=\frac{1}{\tau_{IF}}=\frac{64\pi^{4}}{3h\lambda_{IF}^{3}}\chi\left|\mathop{\mu_{IF}}\limits^{\to} \right|^{2}$$

where *Γ_IF_* denotes the spontaneous radiative rate, *h* is Planck’s constant, $\vec{\mu}_{\mathrm{IF}}$ is dipole moment between *I* and *F*. Consequently, the decay lifetime *τ* is proportional to *λ^3^*. The luminescence decay lifetimes *τ*_1200-corr_ monitoring at 1200 nm is corrected by following:

$$\tau_{1200-corr}=\tau_{1200}\frac{\lambda_{m}}{1200^{3}}$$

where *τ*_1200_ denotes the lifetime before wavelength correction, *λ*_m_ refers to the emission wavelength (720, 780, and 890 nm). Accordingly, the lifetimes are comparatively listed in Table S2.

Table S2. Lifetimes data of 0.2 Cr^3+^ samples monitoring at different emissions.

| Emission (nm) | Lifetime (μs) | *τ_1200-corr_* (μs) |
| --- | --- | --- |
| 780 | 541 | 634 |
| 890 | 89 | 942 |

[1] C.-K. Duan, M. F. Reid, *Curr. Appl. Phys.* **2006**, *6*, 348.

[2] D. Toptygin, *J. Fluoresc.* **2003**, *13*, 201.

Table S3. Bond length data of Ga1-O, Ga4-O, and Ga5-O octahedra.

|  | **Bond**  **length**  **(Å)** | **Average**  **(Å)** |  | **Bond**  **length**  **(Å)** | **Average**  **(Å)** |  | **Bond**  **length**  **(Å)** | **Average**  **(Å)** |
| --- | --- | --- | --- | --- | --- | --- | --- | --- |
| Ga^1^-O | 1.946 | 1.946 | Ga^4^-O | 1.926 | 1.997 | Ga^5^-O | 1.903 | 1.978 |
|  | 1.946 |  |  | 1.926 |  |  | 1.922 |  |
|  | 1.946 |  |  | 1.930 |  |  | 1.922 |  |
|  | 1.946 |  |  | 2.068 |  |  | 2.032 |  |
|  | 1.946 |  |  | 2.068 |  |  | 2.032 |  |
|  | 1.946 |  |  | 2.073 |  |  | 2.054 |  |
